# Differential Expression of Maize and Teosinte microRNAs under Submergence, Drought, and Alternated Stress

**DOI:** 10.3390/plants9101367

**Published:** 2020-10-15

**Authors:** Edgar Baldemar Sepúlveda-García, José Francisco Pulido-Barajas, Ariana Arlene Huerta-Heredia, Julián Mario Peña-Castro, Renyi Liu, Blanca Estela Barrera-Figueroa

**Affiliations:** 1 Laboratorio de Biotecnología Vegetal, Instituto de Biotecnología, Universidad del Papaloapan, Tuxtepec 68301, Mexico; edgarbal31@gmail.com (E.B.S.-G.); julianpc@unpa.edu.mx (J.M.P.-C.); 2 Division de Estudios de Posgrado, Universidad del Papaloapan, Tuxtepec 68301, Mexico; jfpb.leg@gmail.com; 3 Cátedras CONACyT-UNPA, Universidad del Papaloapan, Tuxtepec 68301, Mexico; arianaahuertah@hotmail.com; 4 Center for Agroforestry Mega Data Science, Haixia Institute of Science and Technology, Fujian Agriculture and Forestry University, Fuzhou 350002, China; ryliu@fafu.edu.cn

**Keywords:** hypoxia, submergence, drought, alternated stress, maize, teosinte, microRNAs

## Abstract

Submergence and drought stresses are the main constraints to crop production worldwide. MicroRNAs (miRNAs) are known to play a major role in plant response to various stresses. In this study, we analyzed the expression of maize and teosinte miRNAs by high-throughput sequencing of small RNA libraries in maize and its ancestor teosinte (*Zea mays* ssp. *parviglumis*), under submergence, drought, and alternated stress. We found that the expression patterns of 67 miRNA sequences representing 23 miRNA families in maize and other plants were regulated by submergence or drought. miR159a, miR166b, miR167c, and miR169c were downregulated by submergence in both plants but more severely in maize. miR156k and miR164e were upregulated by drought in teosinte but downregulated in maize. Small RNA profiling of teosinte subject to alternate treatments with drought and submergence revealed that submergence as the first stress attenuated the response to drought, while drought being the first stress did not alter the response to submergence. The miRNAs identified herein, and their potential targets, indicate that control of development, growth, and response to oxidative stress could be crucial for adaptation and that there exists evolutionary divergence between these two subspecies in miRNA response to abiotic stresses.

## 1. Introduction

As a consequence of global warming, hydrological fluctuation events such as excessive rainfall and droughts are common and projected to continue in the future, affecting economic activities [1]. Alterations in water availability in the field, either caused by water surplus or deficit, produce water stress in plants that negatively impacts the growth and productivity of crops worldwide [2].

Flooding affects the properties of soil and the composition of associated microbial communities [3] and reduces the availability of nutrients and flux of oxygen to the plant. This leads to hypoxic stress at the cellular level, especially when the column of water exceeds the length of the stem (i.e., submergence) [4,5]. In response to flooding stress, plants express genes known as hypoxia core genes (HCG) that promote anaerobic metabolism [6]. Additionally, some plants use the escape strategy by redirecting growth to stem elongation to overpass the column of water and maintain the oxygen flux. Plants may also delay growth, flowering, and other processes that are energetically expensive, as part of a strategy known as quiescence to save resources useful to reestablish normal growth when the water level recedes [7]. On the contrary, when water availability is limited, as in the case of drought, plants undergo a similar series of changes to adapt to the adverse conditions, which include stomatal closure with limitations in CO_2_ uptake and reduction in photosynthetic activity, increase in root elongation rate, early or delayed flowering time, and other physiological and morphological changes [8].

Cellular dehydration caused by drought, and hypoxia driven by submergence, trigger a cascade of adaptive responses that are regulated at the molecular level in plants and are directed to maintain vital functions and protect structures from damage. Since cellular protection is a priority during stress, it has been shown that some responses to dehydration and excess water have common molecular effectors acting as nodes in the crosstalk between responses to drought and submergence [9,10,11]. This may have allowed wild plants to evolve tolerance mechanisms to cope with alternate drought and flooding events, which commonly occur consecutively in some regions of the world [12,13].

Maize (*Zea mays* ssp. *mays*) is one of the main dietary cereals in the world [14]. In the United States, the world’s main maize producer, extreme drought and excessive rainfall are the first and second major causes of losses in maize production, respectively, with both affecting yields to a comparable extent [15]. According to archeological, botanical, and genetic evidence, maize was domesticated from its single ancestor teosinte (*Zea mays* ssp. *parviglumis*) around 10,000 years ago [16,17]. It is estimated that maize retained 83% of the nucleotide diversity from its ancestor [18]. During domestication and breeding, maize has been subject to selection for traits that are beneficial to improve feeding quality and yield, while other traits like tolerance to stress could have been lost. For this reason, teosinte is considered a living reservoir of genes and mechanisms that could be of importance to improve tolerance to stress in maize [19,20].

MicroRNAs (miRNAs) have been proposed as promising targets for developing plants with improved tolerance to multiple abiotic stresses [21]. miRNAs are small RNA molecules with pivotal roles in the response to environmental stresses in plants that usually act as negative regulators of gene expression via silencing target genes, which are recognized by sequence complementarity and subsequently targeted for degradation and/or translation inhibition [22]. With the development of new platforms for the high-throughput sequencing of small RNAs, several miRNAs involved in the response to drought or submergence have been described in plants such as *Arabidopsis thaliana* [23,24], *Brachypodium distachyon* [25,26], *Oryza sativa* [27], *Nelumbo nucifera*, [28] and *Zea mays* [29,30].

According to miRBase, 325 mature miRNAs from 174 miRNA precursors have been identified in maize [31], and some of them have been reported to be responsive to drought [32,33], submergence, [34] or waterlogging [35]. For example, miR159, miR164, miR167, miR393, miR408, and miR528 are upregulated by short-term waterlogging in roots of maize lines with high tolerance and suggest the involvement of hormonal control in the response mediated by miRNAs [30]. Another study of two maize lines, with contrasting tolerance to drought, observed the downregulation of miR164 and upregulation of miR159, miR390, and miR398 in the tolerant line compared with the sensitive line [29]. However, it is not clear how miRNAs are regulated in teosinte under submergence or drought stress and whether miRNA response to these stresses is conserved during maize domestication and breeding.

Therefore, this study aimed to identify microRNAs expressed in maize and teosinte under submergence and drought stress in order to analyze the conserved and differential mechanisms of response in these plants. For this, we used high-throughput small RNA sequencing to profile maize and teosinte under submergence, drought, and consecutive drought and submergence stresses. We found a group of miRNAs that were regulated by drought or submergence, including some miRNAs that were differentially regulated in the two species. Analyses of small RNA data from teosinte subject to alternate treatments of drought and submergence indicated that miRNA response to drought was attenuated when submergence was the first stress treatment, but miRNA response to submergence did not change when drought stress was applied first. The potential targets of these miRNAs indicate that control of development, growth, and response to oxidative stress could be crucial for understanding the conservation and divergence of stress tolerance between maize and teosinte.

## 2. Results

### 2.1. Physiological Response of Maize and Teosinte to Submergence and Drought

**Figure 1:**
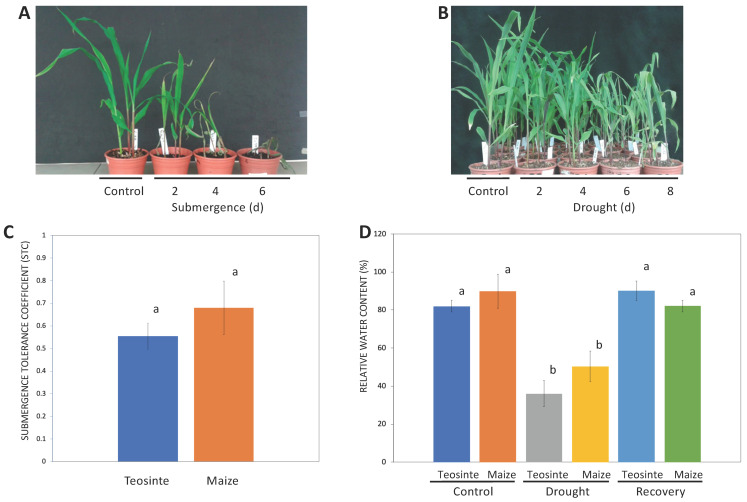
Effects of submergence and drought on maize and teosinte plants. (**A**) Plants exposed to submergence for 2, 4, and 6 days, and (**B**) plants exposed to drought for 2, 4, 6, and 8 days. Response of teosinte and maize plants to (**C**) submergence for 48 h, expressed as submergence tolerance coefficient (STC), and (**D**) drought for 6 days and recovery 24 h after rehydration, expressed as relative water content (RWC) in the leaves. Data are means, with error bars representing ±SD of three biological replicates, each replicate consisting of six plants. Different letters above the error bars indicate statistically significant differences between samples (*p* < 0.05) in a Student’s *t*-test.

With the objective to determine a time period of treatment for RNA collection, maize seedlings with two vegetative leaves and 14 days after sowing (stage V2, 14 DAS) were exposed to submergence for 2, 4, and 6 days (Figure 1A). All treatments induced growth reduction in maize, but plants submerged for 4 or 6 days developed necrotic spots on the leaves, and plants treated for 6 days were not able to recover from the stress. Therefore, a 2 day submergence treatment was selected for subsequent assays. Similarly, drought assays were performed on maize seedlings that were deprived of water until they reached half their initial weight and were maintained under this level of water limitation for 2, 4, 6, and 8 days (Figure 1B).

Drought caused a reduction in the growth of maize plants when compared with control plants, but this reduction was stabilized, which may be explained by the fact that pots were maintained at a constant, intermediate water deficit condition, and not exposed to progressive drought stress. Based on these observations, maize and teosinte plants were exposed to submergence for 2 days and drought for 8 days, and the submergence tolerance coefficient (STC) and relative water content (RWC) were registered for the respective treatments. Submergence affected the growth of maize and teosinte seedlings to a similar extent, since STC was not significantly different (Figure 1C). There were also no significant differences in RWC between maize and teosinte in control, drought, and 24 h of recovery after the stress (Figure 1D). These results suggest that both plants are able to sense and respond to stress by adjusting water status and growth to a similar extent at this stage. This is expected because both maize CML496 and teosinte grow in conditions prevalent in the tropics, where drought, high temperature, and extreme seasonal variations in monthly rainfall are commonly observed. Therefore, this constitutes a solid system to investigate the intra-specific conservation and divergence in the response to stress between maize and its direct ancestor that lead to adaptation in both plants.

### 2.2. Overall Analysis of Small RNA Sequencing Data

The alterations in growth, STC, and RWC observed in maize and teosinte seedlings in the assays suggest that the times and stress intensities can help to study molecular responses in both plants. Accordingly, maize and teosinte seedlings exposed to 2 days of submergence or 6 days of constant drought were selected for profiling the microRNA populations involved in the corresponding responses. In addition, teosinte seedlings exposed to alternated stress of drought-submergence and submergence-drought were also selected.

High-throughput sequencing of small RNA libraries prepared from teosinte and maize under submergence, drought, and alternated stress rendered from 16 to 46 million raw reads (Table 1), with PHRED score above 30 (Figure S1). After removing low-quality reads and adapters, the libraries were analyzed for size distribution of reads, revealing an enrichment of 24 nt sRNAs across the libraries (Figure S2). Reads less than 18 or longer than 35 nt were removed, resulting in a number of clean reads ranging from 14.8 to 41.9 million (Table 1).

On average, 99.7% of the clean reads were mapped to the maize genome. From the reads mapped to the maize genome, less than 3% represented plant miRNAs reported in the miRBase (Table 1). Interestingly, libraries from teosinte showed the lowest percentage of miRNAs (approximately 0.74% for teosinte and approximately 2.19% for maize), suggesting a potential underrepresentation effect caused by the absence of teosinte miRNAs in the miRBase. However, the percentage of teosinte reads that were mapped to the maize genome (approximately 99.75%) was the same as that for maize (approximately 99.77%), indicating that differences are likely due to lower levels of miRNA expression in teosinte. In addition, a reduced abundance of plant miRNAs under submergence stress was observed in both maize and teosinte, and in teosinte treated with drought-submergence stress compared with the corresponding controls (Table 1). After removing low frequency reads and normalization to tags per million (TPM), 178 miRNA sequences in total (118 mapping to maize and 60 to miRNAs of other plants) were expressed across the libraries (Table S1).

### 2.3. Global Changes in Expression of miRNA Sequences in Response to Stress

Further analysis revealed that 164 miRNA sequences (110 from maize and 54 annotated in other plants) were differentially expressed in maize and/or teosinte in response to at least one of the treatments (Table S1).

**Figure 2:**
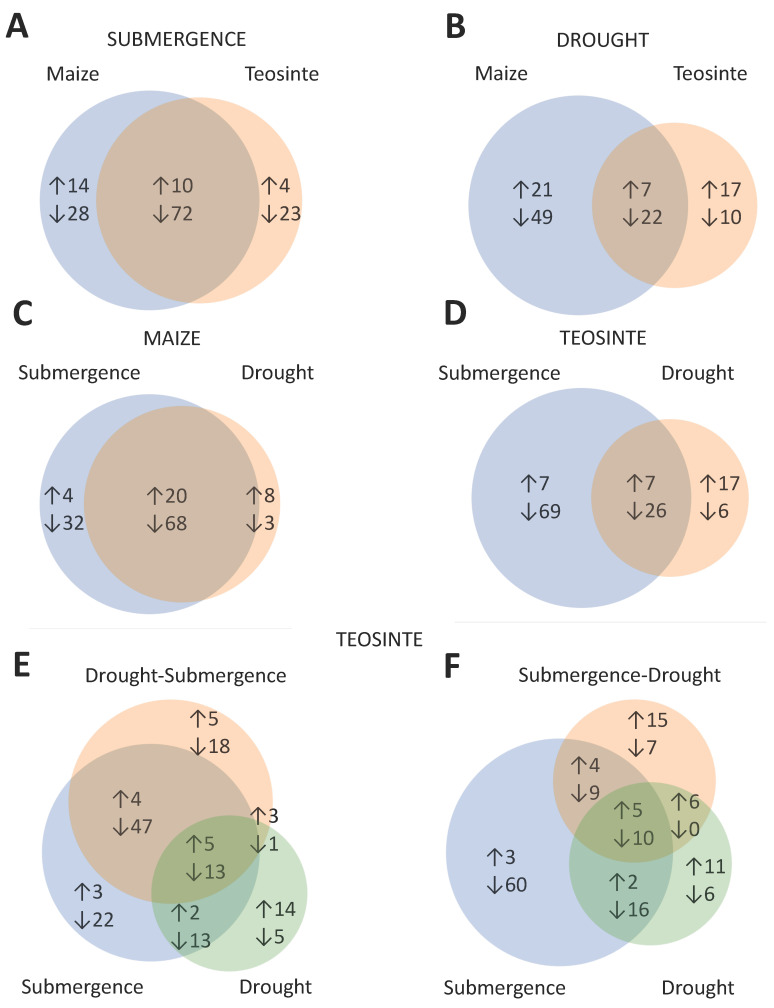
Venn diagrams showing differentially expressed miRNA reads that overlap in maize and teosinte under (**A**) submergence and (**B**) drought treatments. Plant miRNAs overlapping in submergence and drought treatments in (**C**) maize, and (**D**) teosinte. Plant miRNAs overlapping in teosinte under single and alternated treatments of (**E**) drought followed by submergence, and (**F**) submergence followed by drought. Numbers are based on fold change (FC) (log_2_) values (treatment/control). Upward arrows represent upregulated miRNAs (FC ≥ 1) and downward arrows represent downregulated miRNAs (FC ≤ −1).

Regarding submergence stress, maize and teosinte showed common expression profiles of 10 upregulated and 72 downregulated miRNA sequences (Figure 2A). A total of 42 miRNA sequences (14 upregulated and 28 downregulated) were regulated exclusively in maize, while 27 miRNA sequences (4 upregulated and 23 downregulated) were exclusive of teosinte (Figure 2A). A similar trend in the number of miRNAs responsive to stress, with lesser miRNAs being regulated in teosinte than in maize, was observed for drought treatments (Figure 2B). The analysis revealed 70 miRNA sequences (21 upregulated, 49 downregulated) regulated exclusively in maize, while 27 were differentially expressed only in teosinte (17 upregulated, 10 downregulated).

The overlap between submergence and drought responses in maize and teosinte showed that in maize, most of the miRNAs (68 sequences) were downregulated in both submergence and drought (Figure 2C), whereas in teosinte, the number of sequences downregulated exclusively in submergence was the highest (69 sequences; Figure 2D).

In addition, small RNA libraries from teosinte exposed to alternated water stress were analyzed to assess the effects of consecutive treatments of drought and submergence on miRNA expression. Figure 2E shows that 18 miRNA sequences (5 upregulated and 13 downregulated) were differentially expressed in a similar pattern in response to submergence, drought, and alternated drought-submergence treatments. The number of miRNA sequences (69) overlapping between drought-submergence and submergence (representing 63% of total sequences in submergence) was higher compared with only 22 miRNA sequences between drought-submergence and drought (39% of total sequences in drought; Figure 2E), indicating that when submergence is the second stress, it has the strongest effect on miRNA expression.

When submergence is followed by drought, 21 out of 56 miRNA sequences regulated under drought overlapped between submergence-drought and drought treatments (37% of sequences in drought), indicating that when submergence is the first stress, it affects the subsequent response to drought in teosinte (Figure 2F).

### 2.4. Redundancy of miRNA Reads Annotated to miRBASE and Grouping of Representative Sequences

The data showed a high level of redundancy of reads in the libraries annotated to miRBASE, mainly caused by positional variants of mature miRNAs and other sequences matching miRNA precursors. Regrouping of mature miRNAs, positional variants derived from the same mature sequence, and sequences that exactly match those from other plants but with a close similarity to known maize miRNAs (i.e., differing by one or two nucleotides in the 5′ or 3′ ends), rendered 67 representative sequences belonging to 23 miRNA families (Table S2).

### 2.5. Search for Novel miRNAs

In order to identify new variants of known miRNAs in teosinte, a search was performed against the miRBASE, allowing up to two mismatches in the mature or precursor sequences. This rendered 102 polymorphic variants that showed the same expression profiles as those of their corresponding exact sequences (Table S3). However, these sequences failed to provide a perfect hit to the reference genome, which did not allow the analysis of a putative precursor sequence. We also searched for potential novel miRNAs in our datasets and generated several candidates, but after a detailed inspection, they did not comply with the criteria for annotation of novel miRNAs in terms of secondary structure and/or distribution of reads along the potential precursor [36].

### 2.6. Analysis of miRNAs Responsive to Submergence and Drought in Maize and Teosinte

In the group of 67 representative non-redundant miRNA sequences, 13 were identified as responsive to at least one of the stress conditions, with fold change values ranging from −4.39 for the most downregulated miRNA (miR166bd) to 2.17 for the most upregulated miRNA (miR319b) (Table 2). Besides these miRNAs, others showing differential expression such as miR1511, miR2916, miR482, and miR4995 (Table S2), were discarded from the analysis due to failure to hit the maize genome or originating from ribosomal RNA or transposons.

Overall, the grouping of miRNAs presented a clear picture of the stress response. Changes in miRNA expression were more marked in maize than in teosinte for most miRNAs downregulated under submergence, suggesting that maize plants could be more sensitive or reactive to submergence than teosinte in terms of miRNA responses. miRNAs that were specifically downregulated in maize were miR159ab, miR164e, miR166bd, miR167cdeg, miR169cr, miR396cd, and miR398b (Table 2).

In maize, drought stress repressed the expression of five miRNAs (miR156k, miR164e, miR166bd, miR167cdeg, and miR528a). In teosinte, only miR166bd and miR408 were downregulated, while five miRNAs were upregulated under drought (miR156k, miR159ab, miR164e, miR319b, and miR396cd) (Table 2).

### 2.7. Analysis of miRNAs Responsive to Alternated Stress in Teosinte

When plants were exposed to drought-submergence conditions, the miRNA expression profile resembled that of submergence, suggesting that at the end of the drought treatment, plants were not compromised to respond to a submergence event (Table 3). The changes between drought-submergence and submergence stress were limited only to the expression of miR408. This miRNA target gene encodes plastocyanin (PLC) and laccase (LAC) involved in copper homeostasis and the formation of lignin.

When plants were exposed to submergence-drought conditions, most miRNAs were regulated in a way similar to that in plants under drought alone, but the response was attenuated. For example, miR159ab, miR319b, and miR396cd were upregulated in drought as a single stress, but when drought was preceded by submergence, regulation of these miRNAs was maintained within the same trend but at low levels (Table 3). These results suggest that plants are plastic in response to environmental cues and have the capacity to oscillate to adapt to different stresses.

### 2.8. Assessment and Validation of miRNA Expression by Quantitative RT-PCR

**Figure 3:**
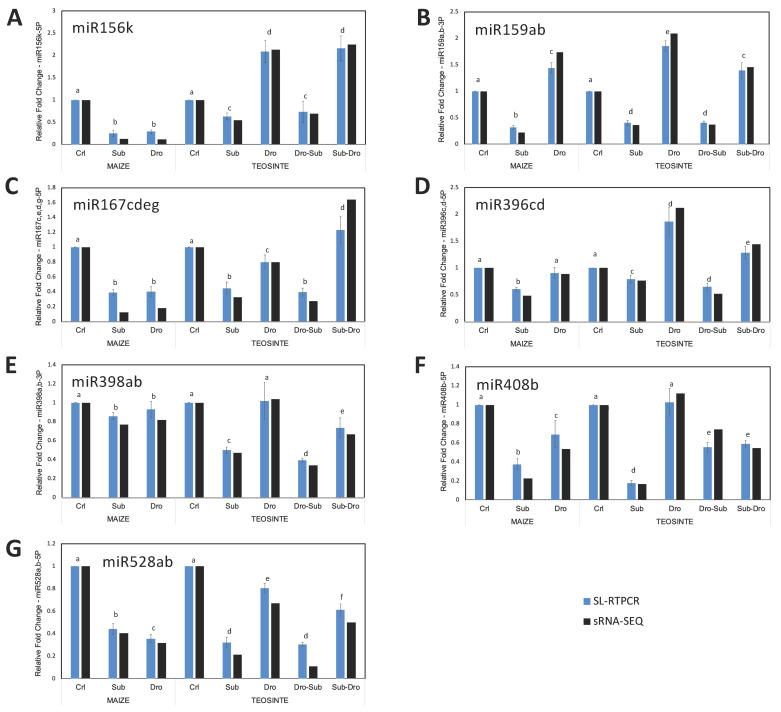
qRT-PCR validation of miRNAs response to submergence, drought, and alternated stress by SL-RTPCR for (**A**) miR156, (**B**) miR159, (**C**) miR167, (**D**) miR396, (**E**) miR398, (**F**) miR408, and (**G**) miR528. Relative fold change values were calculated as ∆∆Ct for SL-RTPCR (blue bars) using miR166c as the constitutive control or as the treatment/control ratio from normalized reads for sRNA-SEQ data (black bars). Results are mean with error bars representing ±SD of three biological replicates, each replicate consisting of a pool of six plants and three technical replicates. Different letters above the error bars indicate statistically significant differences between samples (*p* < 0.05).

Quantitative RT-PCR assays were performed using a modified SL-RTPCR method for seven miRNAs regulated by stress. Based on the fold change values of miRNAs expressed in the libraries, miR166c-5P was selected as a constitutive control for qRT-PCR assays, since it showed intermediate abundance levels and stable regulation in single and alternated stress conditions (Table 2 and Table 3). SL-RTPCR assays showed changes in expression similar to those observed by analysis of sequencing data for miR156k, miR159ab, miR167cdeg, miR396cd, miR398ab, miR408b, and miR528ab (Figure 3A–G).

For example, based on sequencing data, miR167 had a fold-change value of 0.12 and 0.17 in maize under submergence and drought, respectively. The values obtained by SL-RTPCR were 0.39 (±0.03) and 0.40 (±0.06), respectively, which confirms that miR167 is downregulated by these treatments and thus the expression trends for upregulated or downregulated miRNAs were maintained in both methods.

### 2.9. Other Small RNAs

Mitochondria and chloroplasts are the main compartments for energy production that are affected by low water and oxygen availability and may be a source of signals to integrate the cellular response [37,38]. A search was performed in the libraries with the objective of identifying small RNAs potentially originating from maize mitochondria or plastids. For both mitochondria and plastids, the highest number of reads mapped to the subunit 2 of NADH dehydrogenase (Table S4). The reads were derived from a region spanning 53 bp of NADH dehydrogenase subunit 2 with an abundance of up to 820 TPM, comparable to miR408 abundance (Table S2). The second highest number of reads mapped to the trnL-trnF intergenic spacer and tRNA-Phe (trnF) gene from the maize chloroplast. Interestingly, these reads derived from a 32 bp region of the gene did not show a clear expression trend in response to stress; however, a high accumulation of reads in teosinte compared with that in maize was evident, with up to 2273 TPM, comparable to miR398 abundance (Tables S2 and S5). The causes and implications of the existence of these small RNA signatures need to be investigated in the future.

## 3. Discussion

Drought and submergence represent complex conditions that can be dissected into several components such as hypoxia, nutrient, light, osmotic, temperature, and oxidative stress [39]. Understanding how the different components act to integrate the response to stress at all levels of regulation is fundamental to underpin the improvement of tolerance to drought and flooding stress in crops.

miRNAs are a class of small RNAs that act as regulators of gene expression at the transcriptional level. In this study, high-throughput sequencing and analysis of small RNA populations from maize and teosinte under submergence and drought revealed that submergence caused a global reduction in the representation of miRNA expression compared with control or drought conditions. This was observed in roots of wild tomato treated with hypoxia, where only 1.45% of total reads were expressed in hypoxia-treated roots against 2.45% expressed in control roots [40].

Further exploration of the expression of specific miRNA family members allowed the identification of 13 miRNAs with differential expression profiles in response to submergence and drought. The analysis of their expression patterns and functions of their predicted, or previously confirmed targets, allowed the recognition of two main components of the responses to submergence and drought in maize: transcriptional regulation and antioxidant activity.

### 3.1. Transcriptional Regulation

The growth and development processes are driven by hormonal signaling pathways that are fundamental in the response to submergence and drought in plants [41], where transcription factors and miRNAs participate. Several differentially expressed miRNAs identified in this work are known to act over target genes encoding transcription factors such as miR156 (squamosa promoter-binding protein like, SPL) [42,43,44], miR159 (giberellic acid-MYB transcription factor, GAMYB [28,29]), miR164 (NAM-ATAF-CUC domain transcription factor NAC, MYB [45]), miR166 (basic leucine zipper transcription factor, HD-ZIP III [46,47]), miR167 (auxin-responsive factor, ARF [30,35,48]), miR169 (nuclear transcription factor Y subunit alpha, NFYA [49,50,51,52]), miR319 (teosinte branched1/cycloidea/proliferating cell nuclear antigen factor, TCP [53]), and miR396 (growth-regulating factor, GRF [54,55]). Other target genes predicted in this study are listed in Table S6.

Most miRNAs in this category have been related to responses to abiotic stress. miR156 controls developmental transitions and flowering through the activity of their targets (SPL) [42,43,44] which is upregulated in *Arabidopsis* roots under hypoxia and in lotus under submergence [23,28]. Other miRNAs such as miR166 were upregulated in maize roots in response to waterlogging [34] and downregulated in *Arabidopsis* roots in response to hypoxia [23]. miR164 participates in the development of lateral roots [45] and the response to waterlogging in maize roots [30]. miR159 controls petiole elongation and flowering [56] and is upregulated in maize roots exposed to waterlogging [29]. miR167 is upregulated by waterlogging in maize roots [30,34], and downregulated in submerged lotus seedlings [28].

**Figure 4:**
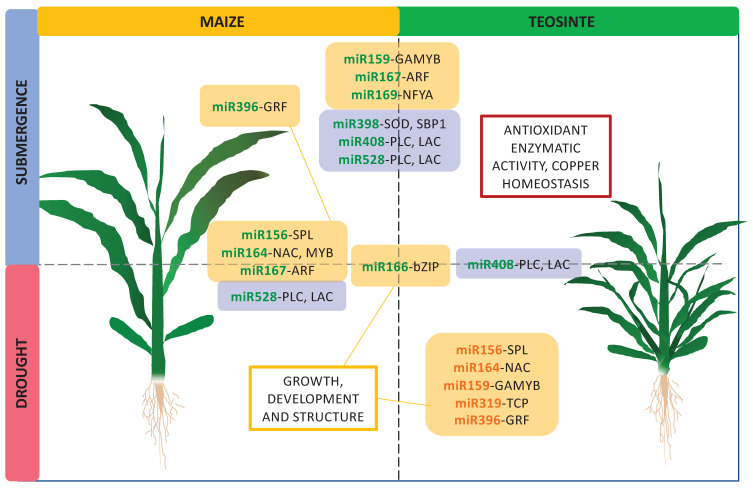
Hypothetical model of the regulation of miRNAs in the response to submergence and drought in maize and teosinte. The importance of antioxidant enzymatic activity, copper homeostasis, and control of growth and development as the main lines of response regulated by miRNAs in both plants is highlighted. Interrupted lines represent overlap between plants and/or treatments. Green, downregulated miRNAs; orange, upregulated miRNAs. GRF: growth-regulating factor; GAMYB: gibberellic acid-MYB transcription factor; ARF: auxin-responsive factor; NFYA: nuclear transcription factor Y subunit alpha; PLC: plastocyanin; LAC: laccase; SOD: superoxide dismutase; SBP1: selenium-binding protein1; bZIP: basic leucine zipper transcription factor; SPL: squamosa promoter-binding protein like; NAC: NAM-ATAF-CUC domain transcription factor; TCP: teosinte branched1/cycloidea/proliferating cell nuclear antigen factor; SBP1: selenium-binding protein 1.

In this study, miRNAs in the category of transcriptional regulation overlapped between maize and teosinte in response to submergence and between submergence and drought in maize (Figure 4), indicating that the control of growth is of central importance for adaptation in maize and teosinte. Interestingly, all these miRNAs were downregulated in the overlaps. Downregulation of this set of miRNAs means that their targets may be released from post transcriptional control, which may activate hormone-responsive genes to initiate elongation (miR159), cell elongation and differentiation (miR167), transition to flowering (miR156), and other responses. In addition, it is possible that changes observed in miRNA expression at the transcriptional level are not only directed to increase growth, but also to mediate feedback in hormonal pathways, or to prepare for reoxygenation or rehydration after stress. Interestingly, the response to drought in teosinte was regulated by five miRNAs that were upregulated (miR156, miR164, miR159, miR319, and miR396). The upregulation of these miRNAs suggests that growth processes may be restricted in teosinte during drought stress (Figure 4).

### 3.2. Antioxidant Activity

Submergence and drought stress result in excessive production of reactive oxygen species (ROS) and efficient mechanisms to cope with oxidative injury are determinants of tolerance [57]. miR398, miR408, and miR528 are included in this category. miR398 target genes encode Cu/Zn superoxide dismutases (CSD1 and CSD2) that act in the defense against toxic ROS in *Arabidopsis* [58]. In *Phaseolus vulgaris,* miR398 is downregulated under drought and submergence [59] and is also downregulated in response to submergence in *Arabidopsis* [60]. We predicted other miR398 targets including a selenium-binding protein (SBP) (Table S6). An SBP was recently reported to be induced in *Arabidopsis* in response to submergence [60] and confirmed as a miR398 target by degradome analysis in maize [61]. In this study, miR398 was downregulated in maize and teosinte under submergence, thereby implicating a potential increase in antioxidant activity.

Other members, such as miR408 and miR528, target genes encoding the cupredoxins PLC and LAC [62,63,64], and other genes controlling circadian clock and flowering time [65,66]. PLC functions as an electron transporter and LAC participates in the formation of lignin by oxidation. miR408 and miR528 have been previously reported to respond to submergence in Lotus seedlings and maize roots [28,30]. In this study, miR408 and miR528 were downregulated in maize and teosinte under submergence, suggesting the maintenance of electron flux, oxidation homeostasis, and lignin synthesis (Figure 4).

### 3.3. Comparison of miRNA Expression in Maize and Teosinte

Previous work demonstrated a high level of conservation of miRNA mature sequences between maize and teosinte [67]. Thus, divergence in tolerance to stress is most likely the result of differences in gene expression between these plants. Overall, in this work maize and teosinte showed similar responses to submergence, with a clear trend toward downregulation of miRNAs involved in the control of growth, development, antioxidant response, and copper homeostasis. However, the response to submergence was more intense in maize than in teosinte for most of the miRNAs responsive to stress, especially for those miRNAs controlling growth. This suggests that maize is more reactive to submergence and responds by releasing the control on their target genes, which in turn may accelerate growth to escape from stress. In teosinte, these responses were less dramatic, suggesting that a finer tuning of expression could be a key for tolerance in this plant. A similar pattern was observed in a study comparing miRNA expression in inbred maize lines with different levels of tolerance to waterlogging, where the stress sensitive line reacted by downregulating most of the miRNAs and induced target genes to accelerate growth. Instead, the tolerant line responded with a moderate level of downregulation, or even upregulation of some miRNAs to repress growth under stress [30].

In the case of drought, maize responded in a similar way as that in submergence by downregulating miRNAs to induce growth, while teosinte upregulated miRNAs to restrict growth and probably save energy resources. Regarding defense against oxidative stress, oxidation homeostasis, and reinforcement of cell structure, both plants maintained the downregulation of miRNAs, suggesting the possibility that their target genes were actively protecting the plants from the oxidative damage caused by stress.

### 3.4. Effects of Alternated Stress on Teosinte

**Figure 5:**
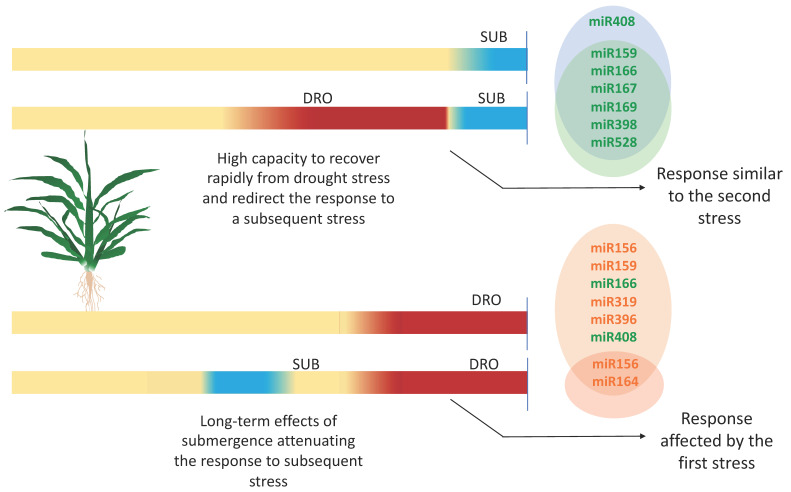
Hypothetical model of the response to alternated stress in teosinte. The influence of the first stress and the consequences in miRNA responsiveness to the second stress are indicated. Green, downregulated miRNAs; orange, upregulated miRNAs.

Alternated events of drought and submergence are common in some regions of the world where plants experience the succession of these events throughout their life cycle. In this study, teosinte plants treated with alternated stress revealed that drought as the first stress had no effect on the capacity to respond to a subsequent event of submergence (Figure 5). In fact, plants treated with alternated stress, and plants treated only with submergence had resembling replicates for most of the miRNAs. This suggests that teosinte was able to rapidly reverse the effects of drought and redirect the response towards submergence, even though drought had induced clear symptoms of stress in plants. The only miRNA that did not respond to submergence as the second stress was miR408, but a functional overlap with miR528 may have supplied the activity of LAC and PLC.

In the opposite order, submergence followed by drought produced an attenuated response to drought. The effects of submergence as the first stress had a long-term influence, considering that between the end of submergence and the beginning of drought, there was a gap of 8 days. This effect also suggests the plastic nature of plants to adapt to adverse factors and oscillate between different stresses.

Research has shown that recovery from submergence represents an additional stress by itself. Upon de-submergence, plants suffer reoxygenation stress conducive to the accumulation of ROS, reillumination stress, dehydration, and senescence [68]. During these processes, hormonal signals, ROS, protective proteins, and other molecular effectors could be induced in plants over a period of time that could be useful to survive any other event of stress such as drought, establishing an overlap between submergence and drought tolerance, as described in rice harboring the SUB1A gene [9].

Several aspects in the involvement of miRNAs in the responses to stress still need to be addressed for constructing a solid system that improves stress tolerance in plants. These include a detailed study of expression dynamics between miRNA and target genes during exposure to combined or alternated stress and during recovery in lines with contrasting tolerance, implications of other factors in the response such as intensity and quality of light during stress, circadian cycle on miRNA expression, plant age, the function of other sRNAs involved, and additional layers of regulation of gene expression. The present study constitutes a base for information that could be useful in further efforts to broaden the knowledge on the roles of miRNAs in stress response and explore miRNAs as tools for improving tolerance to multiple stresses.

## 4. Materials and Methods

### 4.1. Plant Materials

Maize seeds (*Zea mays ssp. mays*) of tropical homozygous line CML496, and teosinte (*Zea mays ssp. parviglumis*) were obtained from Centro de Investigación y de Estudios Avanzados (CINVESTAV) and Instituto Nacional de Investigaciones Forestales, Agrícolas y Pecuarias (INIFAP) seed collections, respectively. The seeds were germinated directly in pots with Sunshine Mix 3 (N: 36 ppm from NO_3_/9 ppm from NH_4_; P: 7 ppm; K: 49 ppm; Ca: 45 ppm; Mg: 30 ppm; S: 39 ppm; B: 0.046 ppm; Cu: 0.01 ppm; Fe: 0.478 ppm; Mn: 0.251 ppm; Mo: 0.035 ppm; Na: 10 ppm; Cl: 8 ppm; Al: 0.48 ppm) (Sun Gro Horticulture Distribution Inc., Agawam, MA, USA), three seeds per pot. In the case of teosinte, the seeds were mechanically scarified to remove the coat before sowing.

### 4.2. Growth Conditions and Treatments

Maize and teosinte plants were grown in a plant growth room at 23 °C and 16 h/8 h light/dark cycle in order to lengthen the vegetative growth phase. The drought stress applied in this study was set to maintain plants at a constant intermediate intensity of drought rather than a progressive drought stress. For drought stress, pots with plants in vegetative stage V2, 12 days after sowing (12 DAS) were water-deprived for 6 days by interrupting water supply until half of their initial weight (water deficit) was reached. Then, water was added in small amounts on a daily basis to constantly maintain the plants in a water deficit condition at half their weight for 6 days. Submergence stress was applied on plants at 26 DAS, with pots in empty 32 cm deep submergence tanks that were filled with filtered tap water to increase the water column by 6 cm every 2 h for 10 h, until the maximum capacity was reached, and plants were fully submerged. The plants were maintained in this condition for 48 h after the first addition of water. For all the treatments, growth conditions were maintained at 23 °C and 16 h/8 h light/dark cycle.

For drought followed by submergence (drought-submergence), teosinte plants (14 DAS) were water-deprived for 6 days followed by 6 days under constant drought stress and then submerged for 48 h as previously described. For submergence followed by drought (submergence-drought), teosinte plants (12 DAS) were submerged for 48 h and removed from the water tank followed by depriving them of water until the plants reached half their initial weight (8 days). Then, small amounts of water were added to maintain a constant water deficit condition for 6 days. The single and alternated stress experiments with maize and teosinte were designed to end simultaneously at 28 DAS. At the end of the experiments, the shoots were immediately collected, frozen in liquid nitrogen, and stored at −80 °C for further processing. All the assays, including control and treatments, were performed with three biological replicates, with each replicate containing six plants.

### 4.3. Assessment of Physiological and Morphological Effects of Stress in Maize and Teosinte

For drought stress, the relative water content (RWC) was measured in the second leaf of maize plants under drought stress and control conditions, as previously described [69]. Briefly, square leaf sections (2 × 2 cm) were excised from plants and weighed to obtain the fresh weight, then submerged in deionized water for 24 h to obtain the turgid weight, and finally dried at 70 °C until constant dry weight was achieved. Then, RWC was calculated as [(fresh weight-dry weight)/(turgid weight-dry weight) × 100].

For submergence stress, the submergence tolerance coefficient (STC) was adapted from the coefficient applied for waterlogging [70]. Briefly, the length of shoots and roots of plants exposed to submergence or control conditions were noted at the end of experiments. The plants were then dried at 70 °C until a constant dry weight was reached. The sums of length and dry weight of plants exposed to the stress and control conditions were used to calculate the STC as [(sum of treated plants)/(sum of control plants)]. RWC and STC measurements were performed in three biological replicates, each consisting of six plants per treatment.

### 4.4. Total RNA Extraction and Construction of Small RNA Libraries

Total RNA was extracted from treated and control samples (three biological replicates, each consisting of a pool of six plants) with TRIzol reagent (Life Technologies, Carlsbad, CA, USA) according to the manufacturer’s instructions. Total RNA was quantified using a Nanodrop ND-1000 Spectrophotometer (Thermo Fisher Scientific Inc., Waltham, MA, USA) and visualized by 1% agarose gel electrophoresis under denaturing conditions. Total RNA from triplicates of the same treatment was pooled (4 μg from each biological replicate in a total of 12 μg per treatment). The small RNA fraction (20–30 nt) was purified from total RNA in a denaturing 15% acrylamide gel. Next, a pre-adenylated adapter (linker1, Integrated DNA Technologies, San Diego, CA, USA) was ligated to the 3’ end of small RNAs with T4 RNA ligase II (New England Biolabs, Ipswich, MA, USA) in the absence of ATP. Then, the RNA adapter Illumina RA5 was ligated to the 5´ end of small RNAs with T4 RNA ligase 1 (Ambion, Austin, TX, USA). The ligated products were used as templates in a cDNA synthesis reaction with the primer RT Bridge and the retrotranscriptase MMuLV (Thermo Fisher Scientific Inc., Waltham, MA, USA), followed by a 15-cycle PCR reaction with Phusion High-Fidelity DNA polymerase (New England Biolabs). The PCR products were purified, quantified, and sequenced in the Illumina HiSEQ2500 at the Genomics Core of the University of California-Riverside (Riverside, CA, USA). Adapters and primers are listed in Table S7.

### 4.5. Bioinformatics Analysis of Small RNA Libraries

Raw sequencing datasets from small RNA libraries were processed with the CLC Genomics Workbench 12 (QIAGEN, Hilden, Germany) to remove adapters, low-quality reads, and reads shorter than 18 nt and longer than 35 nt. Clean reads were mapped to the B73 RefGen_v5 maize genome [71] and classified into the different non-coding RNA categories (rRNA, tRNA, snRNA, snoRNA, lncRNA, and repeats). In addition, clean reads were mapped to the maize chloroplast and mitochondria DNA (B73 RefGen_v4). The clean reads were searched against the plant miRNAs deposited in the miRBASE (Release 22.1) [31], allowing (1) zero mismatches, or (2) up to two mismatches to detect polymorphisms. miRNA sequence counts were normalized as TPM relative to the total clean read counts in the corresponding library. Individual miRNA sequences were then analyzed for differential expression. Fold change values were calculated as log_2_ (normalized counts in treatment/normalized counts in control) (Table S1). Only miRNA sequences with raw counts ≥ 50 in at least one of the libraries were considered for differential expression analysis, and fold change values ≥1 or ≤−1 were considered as upregulated or downregulated, respectively.

miRNA sequences with a high level of similarity were then grouped. All sequences that differed from the mature sequence by one or two nucleotides in the 5′ or 3′ ends were considered positional variants and were then grouped with the mature sequence to constitute a representative miRNA group. The sum of reads and fold change values were recalculated for grouped miRNA sequences (Table S2).

Small RNA sequencing data were deposited into the NCBI/GEO database with accession number GSE155050.

### 4.6. Assessment of miRNA Differential Expression by Quantitative RT-PCR (qRT-PCR)

A modified stem-loop method based on that previously described [72,73] was used to assess miRNA expression. Briefly, total RNA from treated and control plants were treated with DNase I (Thermo Fisher Scientific, Inc, Waltham, MA, USA.). Subsequently, 0.1 μg of treated RNA was used in 10 μL-cDNA synthesis reactions using the TaqMan miRNA reverse transcription kit (Applied Biosystems, Foster City, CA, USA) and miRNA specific stem-loop primers. For quantitative PCR, 20 μL-PCR reactions were prepared using Maxima Probe qPCR Master Mix (Thermo Fisher Scientific Inc, Waltham, MA, USA) with a miRNA-specific direct primer, PCR stem-loop reverse primer, and a universal fluorescein amidite (FAM)-labeled probe, in a PikoReal real-time thermal cycler (Thermo Fisher Scientific Inc.). Differential expression values were calculated using the ΔΔCt method [74] with the use of miR166c as an internal control to normalize the relative abundance of each miRNA. All primers and probes used are listed in Table S7.

### 4.7. Prediction of Target Genes

The psRNATarget analysis server (http://plantgrn.noble.org/psRNATarget/) was used for identification of target genes of miRNAs responsive to submergence and drought [75]. DPMIND [76] was used to search for miRNA targets in nine maize degradomes.

## Figures and Tables

**Table 1:** Summary of small RNA sequencing data from eight maize and teosinte libraries. Distribution of small RNAs in the corresponding non-coding RNA category.

	Maize			Teosinte				
	Control	Submerg	Drought	Control	Submerg	Drought	Drought-Submerg	Submerg-Drought
Raw	16,139,354	26,621,448	31,642,210	20,672,413	28,656,756	29,063,588	17,997,037	46,522,229
Clean	14,897,058	24,568,439	29,744,161	18,955,892	26,296,921	26,960,313	15,760,258	41,915,673
Maize	14,870,947	24,488,359	29,696,341	18,927,434	26,217,672	26,912,777	15,727,757	41,761,974
	99.82%	99.67%	99.84%	99.85%	99.70%	99.82%	99.79%	99.63%
mt	87,401	106,625	134,420	114,741	73,980	105,999	106,451	145,709
	0.59%	0.43%	0.45%	0.61%	0.28%	0.39%	0.68%	0.35%
cp	182,414	452,301	459,718	475,121	444,434	835,675	973,949	1,289,213
	1.22%	1.84%	1.55%	2.51%	1.69%	3.10%	6.18%	3.08%
mRNA	195,418	258,447	342,833	207,230	202,191	256,739	238,774	309,551
	1.31%	1.05%	1.15%	1.09%	0.77%	0.95%	1.52%	0.74%
rRNA	288,257	601,629	778,148	687,038	591,868	588,819	1,056,243	1,240,514
	1.94%	2.45%	2.62%	3.62%	2.25%	2.18%	6.71%	2.97%
tRNA	103,451	161,288	200,294	569,294	480,975	553,939	953,794	1,426,872
	0.70%	0.66%	0.67%	3.00%	1.83%	2.05%	6.06%	3.41%
snRNA	516,999	838,151	1,071,157	576,299	868,349	890,657	421,404	1,220,748
	3.47%	3.42%	3.60%	3.04%	3.31%	3.30%	2.67%	2.92%
snoRNA	105,518	168,088	228,389	141,006	174,443	190,297	111,528	256,675
	0.70%	68.00%	0.77%	0.74%	0.66%	0.70%	0.70%	0.61%
lncRNA	258,027	416,896	519,449	362,527	530,505	528,963	231,375	719,402
	1.73%	1.70%	1.74%	1.91%	2.02%	1.96%	1.47%	1.72%
repeats	2,219,656	3,623,199	4,820,455	5,064,798	5,305,481	5,343,314	4,734,879	9,267,896
	14.92%	14.79%	16.23%	26.75%	20.23%	19.85%	30%	22.19%
Maize	397,473	248,193	774,680	180,091	98,923	310,478	47,836	350,678
miRNAs	2.67%	1.01%	2.60%	0.95%	0.38%	1.15%	0.30%	0.84%
Other	24,837	21,526	39,108	6498	5862	7393	6470	13,887
miRNAs	0.17%	0.09%	0.13%	0.03%	0.02%	0.03%	0.04%	0.03%

Submerg: Submergence; mt: mitochondria; cp: chloroplast; mRNA: messenger RNA; rRNA: ribosomal RNA; tRNA: transfer RNA; snRNA: small nuclear RNA; snoRNA: small nucleolar RNA; lncRNA: long non-coding RNA.

**Table 2:** Representative plant miRNAs responsive to submergence and drought in maize and teosinte.

miRNA Name	Fold Change (FC) ^1^			
	Maize		Teosinte	
	Submergence	Drought	Submergence	Drought
miR156k	−2.94	−2.99	−0.86	1.09
miR159ab	−2.17	0.80	−1.46	1.06
miR164e	−4.24	−1.49	−0.29	1.20
miR166bd	−4.39	−3.79	−2.77	−1.49
miR167cdeg	−2.99	−2.48	−1.62	−0.33
miR169cr	−3.13	−0.95	−1.05	0.11
miR319b	−0.75	0.80	−0.66	2.17
miR396cd	−1.04	−0.17	−0.39	1.08
miR398ab	−0.38	−0.29	−1.09	0.05
miR398b	−2.83	−0.94	−0.69	0.29
miR408	−1.34	−0.51	−1.78	−1.76
miR408b	−2.14	−0.89	−2.57	0.17
miR528ab	−1.30	−1.65	−2.22	−0.57
Constitutive				
miR166c	0.00	−0.01	−0.21	−0.19

^1^ Fold change (treatment/control) values ≥ 1 indicate upregulated miRNAs (orange), and values ≤ −1 indicate downregulated miRNAs (green).

**Table 3:** Representative plant miRNAs responsive to single (submergence or drought) and alternated treatments (drought followed by submergence and submergence followed by drought) in teosinte plants.

miRNA Name	Fold Change (FC) ^1^			
	Submergence	Drought	Drought-Submergence	Submergence-Drought
miR156k	−0.86	1.09	−0.54	1.17
miR159ab	−1.46	1.06	−1.43	0.55
miR164e	−0.29	1.20	0.06	1.09
miR166bd	−2.77	−1.49	−1.95	−0.77
miR167cdeg	−1.62	−0.33	−1.87	0.71
miR169cr	−1.05	0.11	−1.60	0.11
miR319b	−0.66	2.17	−0.41	0.16
miR396cd	−0.39	1.08	−0.94	0.53
miR398ab	−1.09	0.05	−1.56	−0.58
miR398b	−0.69	0.29	−1.92	−0.57
miR408	−1.78	−1.76	−0.88	0.55
miR408b	−2.57	0.17	−0.43	−0.87
miR528ab	−2.22	−0.57	−3.16	−0.99
Constitutive				
miR166c	−0.21	−0.19	−0.27	−0.19

^1^ Fold change (treatment/control) values ≥ 1 indicate upregulated miRNAs (orange), and values ≤ −1 indicate downregulated miRNAs (green).

## Supplementary Materials

The following are available online at https://www.mdpi.com/2223-7747/9/10/1367/s1, Figure S1: PHRED score distribution of small RNA reads in maize and teosinte libraries; Figure S2: Size distribution of small RNA reads in maize and teosinte libraries; Table S1: Known miRNA sequences expressed in maize and teosinte libraries; Table S2: Grouped sequences of known microRNAs expressed in maize and teosinte libraries; Table S3: Sequence variants of microRNAs expressed in maize and teosinte libraries; Table S4: The most abundant reads that mapped to NADH dehydrogenase subunit 2 from the *Zea mays* organelle DNA; Table S5: The most abundant reads that mapped to the trnL-trnF intergenic spacer and tRNA-Phe (trnF) gene from the maize chloroplast; Table S6: List of predicted targets of submergence and drought-responsive miRNAs; Table S7: List of adapters and primers used in this study.
