# Supplementary material for: Differential Expression of Maize and Teosinte microRNAs under Submergence, Drought, and Alternated Stress
Source: Plants (Basel). 2020 Oct 15;9(10):1367. doi: 10.3390/plants9101367 (PMC7650716; doi:10.3390/plants9101367)
Supplement: Supplementary file 1 [file plants-09-01367-s001.zip › Supplementary Materials_proof 2/Table S7.docx]

Supplementary Table S7. List of adapters and primers used in this study.

|  | **Adapters** |
| --- | --- |
| Linker1 (IDT) | rAppCTGTAGGCACCATCAAT/3ddC/ |
| Illumina5 (RA5) | GUUCAGAGUUCUACAGUCCGACGAUC |
|  | **Primers** |
| RT Bridge | CCTTGGCACCCGAGAATTCCAGATTGATGGTGCCTACAG |
| Rev Index1 | CAAGCAGAAGACGGCATACGAGATCGTGATGTGACTGGAGTTCCTTGGCACCCGAGAATTC |
| Rev Index2 | CAAGCAGAAGACGGCATACGAGATACATCGGTGACTGGAGTTCCTTGGCACCCGAGAATTC |
| Rev Index3 | CAAGCAGAAGACGGCATACGAGATGCCTAAGTGACTGGAGTTCCTTGGCACCCGAGAATTC |
| Rev Index4 | CAAGCAGAAGACGGCATACGAGATTGGTCAGTGACTGGAGTTCCTTGGCACCCGAGAATTC |
| Rev Index5 | CAAGCAGAAGACGGCATACGAGATCACTGTGTGACTGGAGTTCCTTGGCACCCGAGAA TTC |
| Rev Index7 | CAAGCAGAAGACGGCATACGAGATGATCTGGTGACTGGAGTTCCTTGGCACCCGAGAATTC |
| miR166c Forward | AGCGAGGAATGTTGTCTGGC |
| miR166c_SL | AAATTGCCCAGGTGGCGCGCAGGGTCCGAGGTCAGAGCCACCTGGGCAATTTCCTCGA |
| miR156k Forward | AGCCATTGACAGAAGAGAGC |
| miR156k_SL | AAATTGCCCAGGTGGCGCGCAGGGTCCGAGGTCAGAGCCACCTGGGCAATTTGTGCTC |
| miR159ab Forward | CTGCGTTTGGATTGAAGGGA |
| miR159ab_SL | AAATTGCCCAGGTGGCGCGCAGGGTCCGAGGTCAGAGCCACCTGGGCAATTTCAGAGC |
| miR167c-e,g Forward | TGACCGTGAAGCTGCCAGCAT |
| miR167c-e,g_SL | AAATTGCCCAGGTGGCGCGCAGGGTCCGAGGTCAGAGCCACCTGGGCAATTTCAGATC |
| miR396ab Forward | GACGATCCACAGGCTTTCTT |
| miR396ab_SL | AAATTGCCCAGGTGGCGCGCAGGGTCCGAGGTCAGAGCCACCTGGGCAATTTCAGTTC |
| miR398ab Forward | AGCAGTGTGTTCTCAGGTCG |
| miR398ab_SL | AAATTGCCCAGGTGGCGCGCAGGGTCCGAGGTCAGAGCCACCTGGGCAATTTCGGGGG |
| miR408b Forward | ATTCCAGGGACGAGGCAGA |
| miR408b_SL | AAATTGCCCAGGTGGCGCGCAGGGTCCGAGGTCAGAGCCACCTGGGCAATTTCCATGC |
| miR528ab Forward | ACGATGGAAGGGGCATGCA |
| miR528ab_SL | AAATTGCCCAGGTGGCGCGCAGGGTCCGAGGTCAGAGCCACCTGGGCAATTTCTCCTC |
| PCR Stem Loop Reverse | GTGGCGCGCAGGGTCCG |
|  | **Probes** |
| Universal probe | 5-FAM/CAGAGCCAC/ZEN/CTGGGCAATTT/3IABkFQ |
