# Supplementary figures and images for: Differential Expression of Maize and Teosinte microRNAs under Submergence, Drought, and Alternated Stress
Source: Plants (Basel). 2020 Oct 15;9(10):1367. doi: 10.3390/plants9101367 (PMC7650716; doi:10.3390/plants9101367)

A

Maize

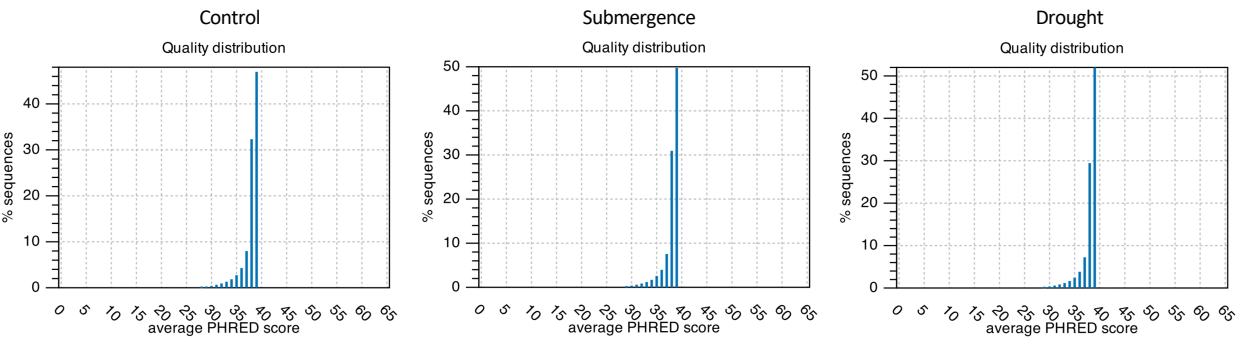

B

Teosinte

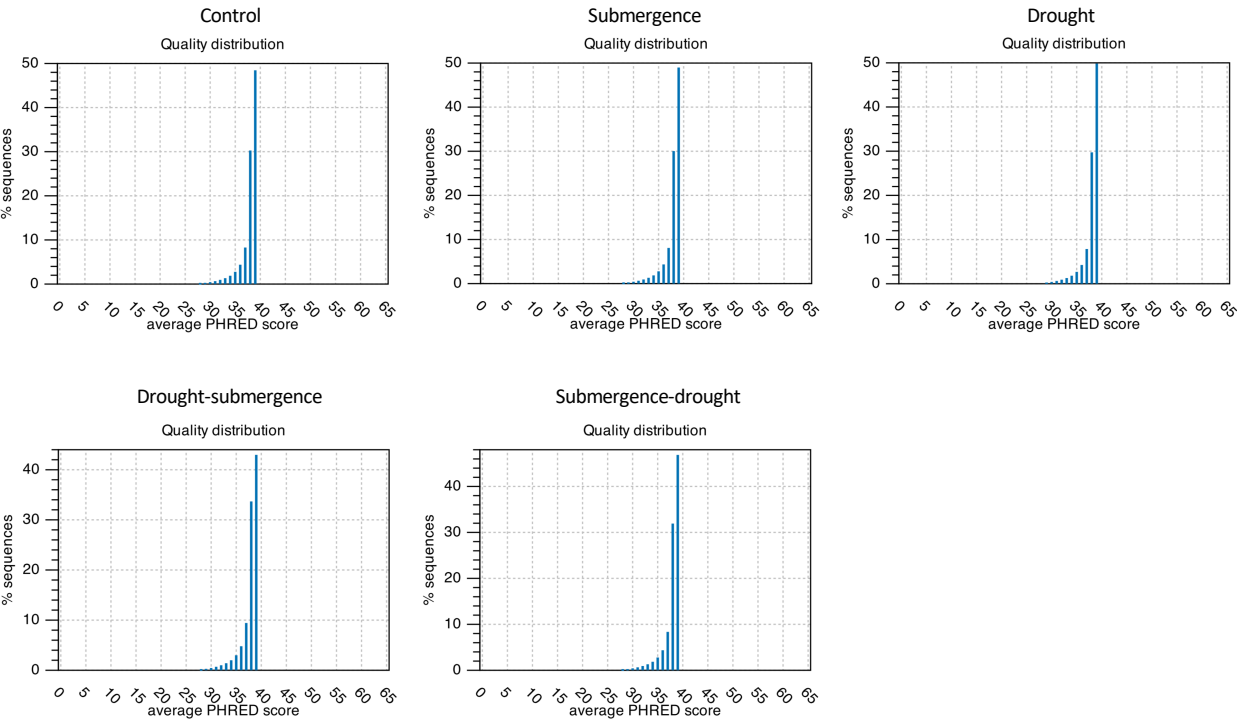

Supplement: Supplementary file 1 [file plants-09-01367-s001.zip › Supplementary Materials_proof 2/Figure S1.pdf]

A

Maize

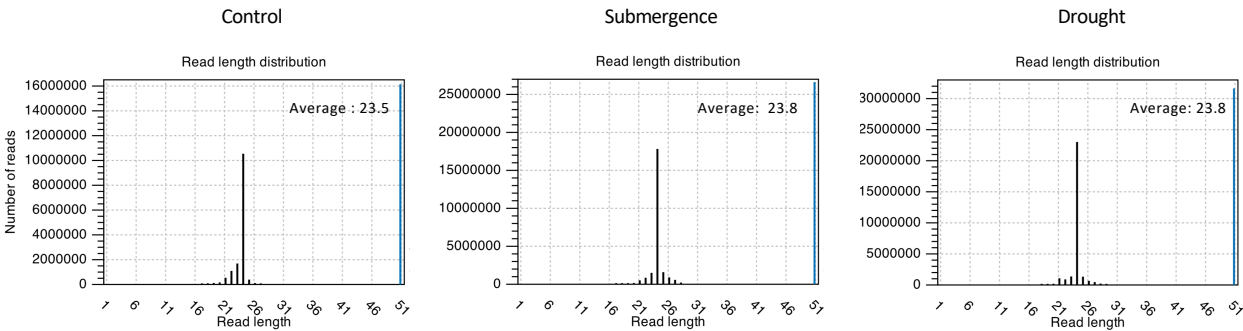

B

Teosinte

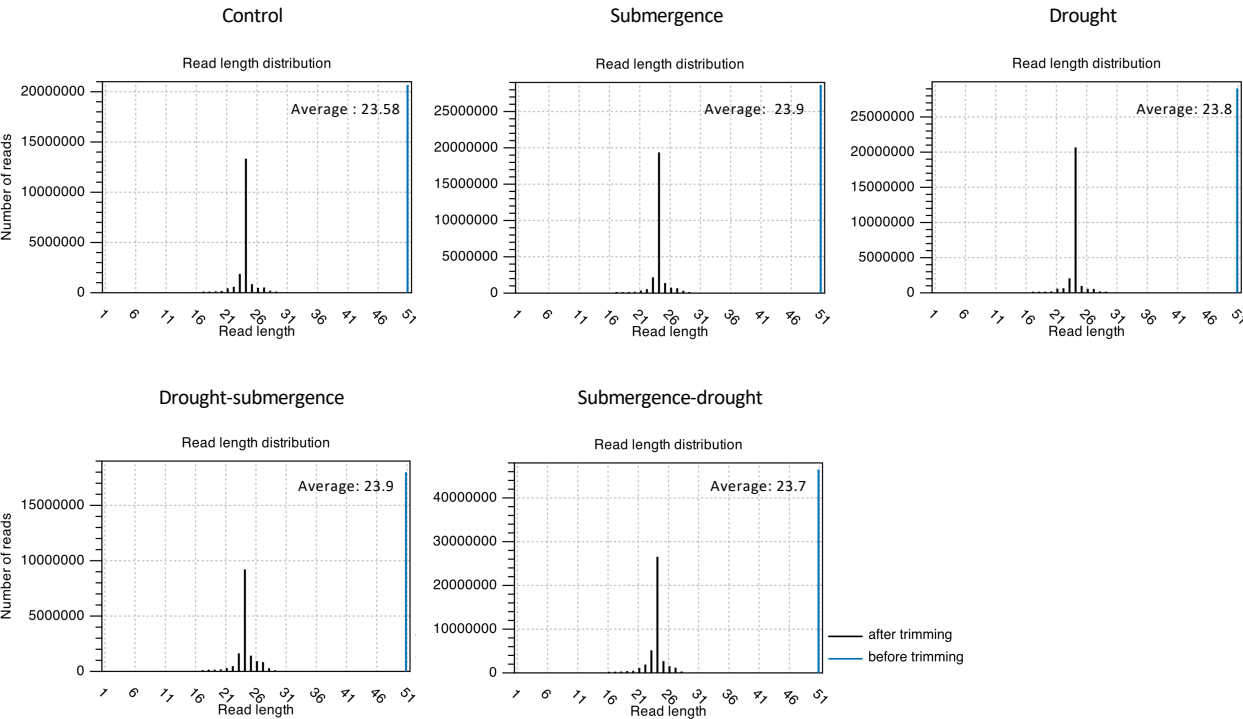

Supplement: Supplementary file 1 [file plants-09-01367-s001.zip › Supplementary Materials_proof 2/Figure S2.pdf]
